# Supplementary material for: Predicting changes in bee assemblages following state transitions at North American dryland ecotones
Source: Sci Rep. 2020 Jan 20;10:708. doi: 10.1038/s41598-020-57553-2 (PMC6971228; doi:10.1038/s41598-020-57553-2)
Supplement: Supplementary file 1 — Supplementary Information. [file 41598_2020_57553_MOESM1_ESM.pdf]

Predicting changes in bee assemblages following state transitions at North American dryland ecotones

Melanie R. Kazenel, Karen W. Wright, Julieta Bettinelli,
Terry L. Griswold, Kenneth D. Whitney, and Jennifer A. Rudgers

Supplementary Information

Includes:
Supplementary Tables S1, S2, S3, S4, S5, S6
Supplementary Figures S1, S2, S3, S4, S5, S6

Supplementary Table S1. Bee species collected during the study. In the Species column, numbers or codes are used as unique identifiers in certain cases where species have not yet been described. Author indicates the authority who described the species.

Family	Genus	Subgenus	Species	Author	Notes
Andrenidae	*Andrena*	*Callandrena*	*ardis*	LaBerge	
Andrenidae	*Andrena*	*Callandrena*	*pecosana*	Cockerell	
Andrenidae	*Andrena*	*Micrandrena*	2		New species
Andrenidae	*Andrena*	*Micrandrena*	*illinoiensis*	Robertson	
Andrenidae	*Andrena*	*Micrandrena*	*primulifrons*	Casad	
Andrenidae	*Andrena*	NA	3		
Andrenidae	*Andrena*	*Plastandrena*	*prunorum*	Cockerell	
Andrenidae	*Andrena*	*Rhaphandrena*	*prima*	Casad	
Andrenidae	*Andrena*	*Tylandrena*	*jessicae*	Cockerell	
Andrenidae	*Andrena*	*Tylandrena*	*mesillae*	Cockerell	
Andrenidae	*Calliopsis*	NA	2		
Andrenidae	*Calliopsis*	*Nomadopsis*	*meliloti*	Cockerell	
Andrenidae	*Calliopsis*	*Perissander*	*anomoptera*	Michener	
Andrenidae	*Calliopsis*	*Verbenapis*	*verbenae*	Cockerell & Porter	
Andrenidae	*Macrotera*	*Macroteropsis*	*latior*	(Cockerell)	
Andrenidae	*Macrotera*	*Macroteropsis*	*magniceps*	(Timberlake)	
Andrenidae	*Macrotera*	*Macroteropsis*	*portalis*	(Timberlake)	
Andrenidae	*Perdita*	*Cockerellia*	*albipennis heliophila*	Cresson	
Andrenidae	*Perdita*	*Cockerellia*	*coreopsidis kansensis*	Timberlake	
Andrenidae	*Perdita*	*Cockerellia*	*verbesinae*	Cockerell	
Andrenidae	*Perdita*	*Epimacrotera*	*diversa*	Timberlake	
Andrenidae	*Perdita*	*Hexaperdita*	4		
Andrenidae	*Perdita*	*Hexaperdita*	aff. *heterotheceae*		
Andrenidae	*Perdita*	*Hexaperdita*	*callicerata*	Cockerell	
Andrenidae	*Perdita*	*Hexaperdita*	*cara*	Timberlake	
Andrenidae	*Perdita*	*Hexaperdita*	*ignota ignota*	Cockerell	
Andrenidae	*Perdita*	NA	10		New species
Andrenidae	*Perdita*	NA	14		
Andrenidae	*Perdita*	NA	15		
Andrenidae	*Perdita*	NA	16		
Andrenidae	*Perdita*	NA	17		
Andrenidae	*Perdita*	NA	18		
Andrenidae	*Perdita*	NA	19		
Andrenidae	*Perdita*	NA	20		
Andrenidae	*Perdita*	NA	21		
Andrenidae	*Perdita*	NA	33		
Andrenidae	*Perdita*	*Pentaperdita*	3		
Andrenidae	*Perdita*	*Pentaperdita*	aff. *albovittata*		
Andrenidae	*Perdita*	*Pentaperdita*	*albovittata*	Cockerell	
Andrenidae	*Perdita*	*Pentaperdita*	*bradleyana*	Timberlake	
Andrenidae	*Perdita*	*Perdita*	2		Sphaeralceae group
Andrenidae	*Perdita*	*Perdita*	5		Sphaeralceae group
Andrenidae	*Perdita*	*Perdita*	6		New species, Octomaculata group
Andrenidae	*Perdita*	*Perdita*	11		New species, Octomaculata group
Andrenidae	*Perdita*	*Perdita*	12		New species, Octomaculata group
Andrenidae	*Perdita*	*Perdita*	13		Chamaesarachae group
Andrenidae	*Perdita*	*Perdita*	31		New species, Octomaculata group
Andrenidae	*Perdita*	*Perdita*	32		New species
Andrenidae	*Perdita*	*Perdita*	36		New species
Andrenidae	*Perdita*	*Perdita*	50		Sphaeralceae group
Andrenidae	*Perdita*	*Perdita*	aff. *cuspidata*		

Family	Genus	Subgenus	Species	Author	Notes
Andrenidae	*Perdita*	*Perdita*	aff. *laticincta*		
Andrenidae	*Perdita*	*Perdita*	aff. *missionis*		
Andrenidae	*Perdita*	*Perdita*	*affinis*	Cresson	
Andrenidae	*Perdita*	*Perdita*	*aperta*	Timberlake	
Andrenidae	*Perdita*	*Perdita*	*aridella*	Timberlake	
Andrenidae	*Perdita*	*Perdita*	*austini*	Cockerell	
Andrenidae	*Perdita*	*Perdita*	*confusa*	Timberlake	
Andrenidae	*Perdita*	*Perdita*	*drymariae*	Timberlake	
Andrenidae	*Perdita*	*Perdita*	*fallax*	Cockerell	
Andrenidae	*Perdita*	*Perdita*	*gutierreziae*	Cockerell	
Andrenidae	*Perdita*	*Perdita*	*lenis*	Timberlake	
Andrenidae	*Perdita*	*Perdita*	*luteola*	Cockerell	
Andrenidae	*Perdita*	*Perdita*	*rectangulata*	Cockerell	
Andrenidae	*Perdita*	*Perdita*	*semicaerulea*	Cockerell	
Andrenidae	*Perdita*	*Perdita*	*sphaeralceae alticola*	Cockerell	
Andrenidae	*Perdita*	*Perdita*	*trinotata*	Timberlake	
Andrenidae	*Perdita*	*Perditella*	*larreae*	Cockerell	
Andrenidae	*Perdita*	*Perditella*	*marcialis*	Cockerell	
Andrenidae	*Protandrena*	*Heterosarus*	1		
Andrenidae	*Protandrena*	*Heterosarus*	2		
Andrenidae	*Protandrena*	*Heterosarus*	3		
Andrenidae	*Protoxaea*	NA	*gloriosa*	(Fox)	
Andrenidae	*Pseudopanurgus*	NA	*fraterculus fraterculus*	(Cockerell)	
Apidae	*Anthophora*	*Anthophoroides*	*californica*	Cresson	
Apidae	*Anthophora*	*Heliophila*	*petrophila*	Cockerell	
Apidae	*Anthophora*	*Heliophila*	*phenax*	(Cockerell)	
Apidae	*Anthophora*	*Lophanthophora*	*affabilis*	Cresson	
Apidae	*Anthophora*	*Lophanthophora*	*coptognatha*	Timberlake	
Apidae	*Anthophora*	*Lophanthophora*	*neglecta*	Timberlake & Cockerell	
Apidae	*Anthophora*	*Lophanthophora*	*porterae*	Cockerell	
Apidae	*Anthophora*	*Mystacanthophora*	*montana*	Cresson	
Apidae	*Anthophora*	*Mystacanthophora*	*urbana*	Cresson	
Apidae	*Anthophora*	NA	n. sp.		Author: Brooks, manuscript in prep
Apidae	*Anthophora*	*Paramegilla*	*centriformis*	Cresson	
Apidae	*Anthophora*	*Pyganthophora*	*lesquerellae*	(Cockerell)	
Apidae	*Anthophorula*	*Anthophorisca*	*pygmaea*	(Cresson)	
Apidae	*Anthophorula*	*Anthophorula*	*compactula*	Cockerell	
Apidae	*Anthophorula*	*Anthophorula*	*completa*	(Cockerell)	
Apidae	*Apis*	*Apis*	*mellifera*	Linnaeus	
Apidae	*Bombus*	*Thoracobombus*	*pensylvanicus*	(DeGeer)	
Apidae	*Centris*	*Paracentris*	*atripes*	Mocsary	
Apidae	*Centris*	*Paracentris*	*caesalpiniae*	Cockerell	
Apidae	*Centris*	*paracentris*	*lanosa*	Cresson	
Apidae	*Centris*	*paracentris*	*zacateca*	Snelling	
Apidae	*Ceratina*	*Zadontomerus*	*nanula*	Cockerell	
Apidae	*Diadasia*	NA	*australis*	(Cresson)	
Apidae	*Diadasia*	NA	*diminuta*	(Cresson)	
Apidae	*Diadasia*	NA	*enavata*	(Cresson)	
Apidae	*Diadasia*	NA	*megamorpha*	Cockerell	
Apidae	*Diadasia*	NA	*ochracea*	(Cockerell)	
Apidae	*Diadasia*	NA	*rinconis*	Cockerell	
Apidae	*Epeolus*	NA	aff. *americanus*		
Apidae	*Epeolus*	NA	*brumleyi*	Onuferko	
Apidae	*Epeolus*	NA	*compactus*	Cresson	
Apidae	*Epeolus*	NA	*mesillae*	(Cockerell)	
Apidae	*Epeolus*	NA	*minimus*	(Robertson)	
Apidae	*Ericrocis*	NA	*lata*	(Cresson)	
Apidae	*Eucera*	*Peponapis*	*pruinosa*	(Say)	
Apidae	*Eucera*	*Synhalonia*	aff. *albescens*		

Family	Genus	Subgenus	Species	Author	Notes
Apidae	*Eucera*	*Synhalonia*	*illinoensis*	(Robertson)	
Apidae	*Eucera*	*Synhalonia*	*lycii*	(Cockerell)	
Apidae	*Eucera*	*Synhalonia*	*territella*	(Cockerell)	
Apidae	*Eucera*	*Tetraloniella*	*albata*	(Cresson)	
Apidae	*Eucera*	*Tetraloniella*	*eriocarpi*	(Cockerell)	
Apidae	*Eucera*	*Tetraloniella*	*silacea?*	LaBerge	Unconfirmed identification
Apidae	*Exomalopsis*	*Phanomalopsis*	*solani*	Cockerell	
Apidae	*Habropoda*	NA	*morrisoni*	(Cresson)	
Apidae	*Habropoda*	NA	*pallida*	(Timberlake)	
Apidae	*Holcopasites*	NA	*calliopsidis*	(Linsley)	
Apidae	*Holcopasites*	NA	*pulchellus*	(Cresson)	
Apidae	*Martinapis*	NA	*luteicornis*	(Cockerell)	
Apidae	*Melecta*	*Melecta*	*alexanderi*	Griswold & Parker	
Apidae	*Melecta*	*Melecta*	*bohartorum*	Linsley	
Apidae	*Melecta*	*Melecta*	*pacifica*	Cresson	
Apidae	*Melissodes*	*Callimelissodes*	*coloradensis*	Cresson	
Apidae	*Melissodes*	*Eumelissodes*	*agilis*	Cresson	
Apidae	*Melissodes*	*Eumelissodes*	*bimatris*	LaBerge	
Apidae	*Melissodes*	*Eumelissodes*	*coreopsis*	Robertson	
Apidae	*Melissodes*	*Eumelissodes*	*fasciatella*	LaBerge	
Apidae	*Melissodes*	*Eumelissodes*	*menuachus*	LaBerge	
Apidae	*Melissodes*	*Eumelissodes*	*montanus*	Cresson	
Apidae	*Melissodes*	*Eumelissodes*	*pallidisignatus*	Cockerell	
Apidae	*Melissodes*	*Eumelissodes*	*perpolitus*	LaBerge	
Apidae	*Melissodes*	*Eumelissodes*	*semilupinus*	Cockerell	
Apidae	*Melissodes*	*Eumelissodes*	*snowii*	Cresson	
Apidae	*Melissodes*	*Eumelissodes*	*subagilis*	Cockerell	
Apidae	*Melissodes*	*Eumelissodes*	*submenuacha*	Cockerell	
Apidae	*Melissodes*	*Eumelissodes*	*tristis*	Cockerell	
Apidae	*Melissodes*	*Eumelissodes*	*verbesinarum*	Cockerell	
Apidae	*Melissodes*	*Melissodes*	*communis communis*	Cresson	
Apidae	*Melissodes*	*Melissodes*	*gilensis*	Cockerell	
Apidae	*Melissodes*	*Melissodes*	*paroselae*	Cockerell	
Apidae	*Melissodes*	*Melissodes*	*thelypodii thelypodii*	Cockerell	
Apidae	*Neolarra*	NA	*vigilans*	(Cockerell)	
Apidae	*Nomada*	NA	spp.		All Nomada were grouped together, as they could not be identified to species.
Apidae	*Svastra*	*Anthedonia*	*compta*	(Cresson)	
Apidae	*Svastra*	*Epimelissodes*	*helianthelli*	(Cockerell)	
Apidae	*Svastra*	*Epimelissodes*	*machaerantherae*	(Cockerell)	
Apidae	*Svastra*	*Epimelissodes*	*obliqua*	(Say)	
Apidae	*Svastra*	*Epimelissodes*	*sabinensis*	(Cockerell)	
Apidae	*Townsendiella*	NA	*pulchra*	Crawford	
Apidae	*Triepeolus*	NA	5		
Apidae	*Triepeolus*	NA	9		
Apidae	*Triepeolus*	NA	*grandis*	(Friese)	
Apidae	*Triepeolus*	NA	*norae*	Cockerell	
Apidae	*Xeromelecta*	NA	*interrupta*	(Cresson)	
Apidae	*Xylocopa*	NA	*californica*	Cresson	
Apidae	*Zacosmia*	NA	*maculata*	(Cresson)	
Colletidae	*Caupolicana*	NA	*yarrowi*	(Cresson)	
Colletidae	*Colletes*	NA	*birkmanni*	Swenk	
Colletidae	*Colletes*	NA	*clypeonitens*	Swenk	
Colletidae	*Colletes*	NA	*compactus*	Cresson	
Colletidae	*Colletes*	NA	*covilleae*	Timberlake	
Colletidae	*Colletes*	NA	*phaceliae*	Cockerell	

Family	Genus	Subgenus	Species	Author	Notes
Colletidae	*Colletes*	NA	*salicicola*	Cockerell	
Colletidae	*Colletes*	NA	*scopiventer*	Swenk	
Colletidae	*Colletes*	NA	*sphaeralceae*	Timberlake	
Colletidae	*Colletes*	NA	*wootoni*	Cockerell	
Colletidae	*Hylaeus*	*Paraprosopis*	1		
Colletidae	*Hylaeus*	*Paraprosopis*	2		
Colletidae	*Hylaeus*	*Paraprosopis*	*asininus*	(Cockerell & Casad)	
Colletidae	*Hylaeus*	*Prosopis*	*episcopelis coquilletti*	(Cockerell)	
Halictidae	*Agapostemon*	NA	*angelicus*	Cockerell	
Halictidae	*Agapostemon*	NA	*melliventris*	Cresson	
Halictidae	*Agapostemon*	NA	*obliquus*	(Provancher)	
Halictidae	*Agapostemon*	NA	*splendens*	(Lepeletier)	
Halictidae	*Agapostemon*	NA	*virescens*	(Fabricius)	
Halictidae	*Augochlorella*	NA	*aurata*	(Smith)	
Halictidae	*Conanthalictus*	NA	*conanthi*	(Cockerell)	
Halictidae	*Dufourea*	NA	*pulchricornis*	(Cockerell)	
Halictidae	*Dufourea*	NA	*vernalis*	Timberlake	
Halictidae	*Halictus*	NA	*ligatus*	Say	
Halictidae	*Halictus*	NA	*tripartitus*	Cockerell	
Halictidae	*Lasioglossum*	*Afrodialictus*	*lampronotum*	(Cameron)	
Halictidae	*Lasioglossum*	*Dialictus*	2		
Halictidae	*Lasioglossum*	*Dialictus*	4		
Halictidae	*Lasioglossum*	*Dialictus*	5		
Halictidae	*Lasioglossum*	*Dialictus*	7		
Halictidae	*Lasioglossum*	*Dialictus*	8		
Halictidae	*Lasioglossum*	*Dialictus*	11		
Halictidae	*Lasioglossum*	*Dialictus*	12		
Halictidae	*Lasioglossum*	*Dialictus*	13		
Halictidae	*Lasioglossum*	*Dialictus*	19		
Halictidae	*Lasioglossum*	*Dialictus*	20		
Halictidae	*Lasioglossum*	*Dialictus*	21		
Halictidae	*Lasioglossum*	*Dialictus*	30		
Halictidae	*Lasioglossum*	*Dialictus*	32		
Halictidae	*Lasioglossum*	*Dialictus*	33		
Halictidae	*Lasioglossum*	*Dialictus*	34		
Halictidae	*Lasioglossum*	*Dialictus*	35		
Halictidae	*Lasioglossum*	*Dialictus*	36		
Halictidae	*Lasioglossum*	*Dialictus*	37		
Halictidae	*Lasioglossum*	*Dialictus*	38		
Halictidae	*Lasioglossum*	*Dialictus*	39		
Halictidae	*Lasioglossum*	*Dialictus*	40		
Halictidae	*Lasioglossum*	*Dialictus*	42		
Halictidae	*Lasioglossum*	*Dialictus*	46		
Halictidae	*Lasioglossum*	*Dialictus*	47		
Halictidae	*Lasioglossum*	*Dialictus*	48		
Halictidae	*Lasioglossum*	*Dialictus*	aff. *pervarum*		
Halictidae	*Lasioglossum*	*Dialictus*	cf. *coactum*		
Halictidae	*Lasioglossum*	*Dialictus*	*comulum*	Michener	
Halictidae	*Lasioglossum*	*Dialictus*	*eophilum*	(Ellis)	
Halictidae	*Lasioglossum*	*Dialictus*	*hudsoniellum*	(Cockerell)	
Halictidae	*Lasioglossum*	*Dialictus*	*microlepoides*	(Ellis)	
Halictidae	*Lasioglossum*	*Dialictus*	n. sp.		New species
Halictidae	*Lasioglossum*	*Dialictus*	*semicaeruleum*	(Cockerell)	
Halictidae	*Lasioglossum*	*Evylaeus*	1		
Halictidae	*Lasioglossum*	*Lasioglossum*	*morrilli*	(Cockerell)	
Halictidae	*Lasioglossum*	*Lasioglossum*	*sisymbrii*	(Cockerell)	
Halictidae	*Lasioglossum*	*Sphecodogastra*	*lusorium*	(Cresson)	
Halictidae	*Nomia*	*Acunomia*	*tetrazonata uvaldensis*	Cockerell	
Halictidae	*Nomia*	*Epinomia*	*triangulifera*	Vachal	
Halictidae	*Sphecodes*	NA	1		

Family	Genus	Subgenus	Species	Author	Notes
Halictidae	*Sphecodes*	NA	2		
Halictidae	*Sphecodes*	NA	4		
Halictidae	*Sphecodes*	NA	5		
Halictidae	*Sphecodes*	NA	6		
Halictidae	*Sphecodosoma*	NA	*dicksoni*	(Timberlake)	
Megachilidae	*Anthidium*	*Anthidium*	*cockerelli*	Schwarz	
Megachilidae	*Anthidium*	*Anthidium*	*emarginatum*	(Say)	
Megachilidae	*Anthidium*	*Anthidium*	*maculosum*	Cresson	
Megachilidae	*Anthidium*	*Anthidium*	*palmarum*	Cockerell	
Megachilidae	*Anthidium*	*Anthidium*	*porterae*	Cockerell	
Megachilidae	*Anthidium*	*Anthidium*	*schwarzi*	Gonzalez & Griswold	
Megachilidae	*Ashmeadiella*	*Arogochila*	*erema*	Michener	
Megachilidae	*Ashmeadiella*	*Ashmeadiella*	*bigeloveae*	(Cockerell)	
Megachilidae	*Ashmeadiella*	*Ashmeadiella*	*bucconis*	(Say)	
Megachilidae	*Ashmeadiella*	*Ashmeadiella*	*cactorum*	(Cockerell)	
Megachilidae	*Ashmeadiella*	*Ashmeadiella*	*difugita*	Michener	
Megachilidae	*Ashmeadiella*	*Ashmeadiella*	*gillettei*	Titus	
Megachilidae	*Ashmeadiella*	*Ashmeadiella*	*meliloti*	(Cockerell)	
Megachilidae	*Ashmeadiella*	*Ashmeadiella*	*occipitalis*	Michener	
Megachilidae	*Ashmeadiella*	*Ashmeadiella*	*opuntiae*	(Cockerell)	
Megachilidae	*Ashmeadiella*	*Ashmeadiella*	*vandykiella*	Michener	
Megachilidae	*Ashmeadiella*	*Isosmia*	*rubrella*	(Michener)	
Megachilidae	*Atoposmia*	*Eremosmia*	aff. *daleae*		New species
Megachilidae	*Atoposmia*	*Eremosmia*	aff. *daleae* 2		New species
Megachilidae	*Coelioxys*	*Xerocoelioxys*	*hirsutissima*	Cockerell	
Megachilidae	*Coelioxys*	*Xerocoelioxys*	*mesae*	Cockerell	
Megachilidae	*Dianthidium*	*Dianthidium*	1		New species
Megachilidae	*Dianthidium*	*Dianthidium*	*concinnum*	(Cresson)	
Megachilidae	*Dianthidium*	*Dianthidium*	*heterulkei*	Schwarz	
Megachilidae	*Dianthidium*	*Dianthidium*	*implicatum*	Timberlake	
Megachilidae	*Dianthidium*	*Dianthidium*	*parvum*	(Cresson)	
Megachilidae	*Dioxys*	NA	aff. *pomonae*		
Megachilidae	*Dioxys*	NA	*pacificus*	Cockerell	
Megachilidae	*Dioxys*	NA	*productus*	(Cresson)	
Megachilidae	*Hoplitis*	*Alcidamea*	*biscutellae*	(Cockerell)	
Megachilidae	*Hoplitis*	*Alcidamea*	*grinnelli*	(Cockerell)	
Megachilidae	*Hoplitis*	*Proteriades*	aff. *torchioi*		
Megachilidae	*Hoplitis*	*Proteriades*	*zuni*	(Parker)	
Megachilidae	*Lithurgopsis*	NA	*apicalis*	(Cresson)	
Megachilidae	*Megachile*	*Argyropile*	*parallela*	Smith	
Megachilidae	*Megachile*	*Argyropile*	*townsendiana*	Cockerell	
Megachilidae	*Megachile*	*Chelostomoides*	*lobatifrons*	Cockerell	
Megachilidae	*Megachile*	*Chelostomoides*	*prosopidis*	Cockerell	
Megachilidae	*Megachile*	*Chelostomoides*	*spinotulata*	Mitchell	
Megachilidae	*Megachile*	*Litomegachile*	*brevis*	Say	
Megachilidae	*Megachile*	*Litomegachile*	*lippiae*	Cockerell	
Megachilidae	*Megachile*	*Megachile*	*montivaga*	Cresson	
Megachilidae	*Megachile*	*Megachiloides*	aff. *dakotensis*		
Megachilidae	*Megachile*	*Megachiloides*	*casadae*	Cockerell	
Megachilidae	*Megachile*	*Megachiloides*	*fucata*	Mitchell	
Megachilidae	*Megachile*	*Megachiloides*	*melanderi*	Mitchell	
Megachilidae	*Megachile*	*Megachiloides*	*mucorosa*	Cockerell	
Megachilidae	*Megachile*	*Megachiloides*	*sublaurita*	Mitchell	
Megachilidae	*Megachile*	*Megachiloides*	*umatillensis*	(Mitchell)	
Megachilidae	*Megachile*	*Megachiloides*	*xerophila*	Cockerell	
Megachilidae	*Megachile*	*Pseudocentron*	*sidalceae*	Cockerell	
Megachilidae	*Megachile*	*Sayapis*	*inimica inimica*	Cresson	
Megachilidae	*Megachile*	*Sayapis*	*policaris*	Say	
Megachilidae	*Osmia*	*Helicosmia*	*coloradensis*	Cresson	
Megachilidae	*Osmia*	*Melanosmia*	1		

Family	Genus	Subgenus	Species	Author	Notes
Megachilidae	*Osmia*	*Melanosmia*	2		
Megachilidae	*Osmia*	*Melanosmia*	aff. *enixa*		New species
Megachilidae	*Osmia*	*Melanosmia*	*cerasi*	Cockerell	
Megachilidae	*Osmia*	*Melanosmia*	*cordata*	Robertson	
Megachilidae	*Osmia*	*Melanosmia*	*gaudiosa*	Cockerell	
Megachilidae	*Osmia*	*Melanosmia*	*integra*	Cresson	
Megachilidae	*Osmia*	*Melanosmia*	*liogastra*	Cockerell	
Megachilidae	*Osmia*	*Melanosmia*	*phenax*	Cockerell	
Megachilidae	*Osmia*	*Melanosmia*	*prunorum*	Cockerell	
Megachilidae	*Osmia*	*Melanosmia*	*trevoris*	Cockerell	
Megachilidae	*Osmia*	*Melanosmia*	*watsoni*	Cockerell	
Megachilidae	*Osmia*	*Trichinosmia*	*latisulcata*	Michener	
Megachilidae	*Stelis*	*Stelis*	*imperialis*	Parker & Griswold	
Megachilidae	*Stelis*	*Stelis*	*palmarum*	Timberlake	
Megachilidae	*Stelis*	*Stelis*	*robertsoni*	Timberlake	
Megachilidae	*Trachusa*	*Heteranthidium*	*larreae*	(Cockerell)	
Mellitidae	*Hesperapis*	*Amblyapis*	*larreae*	Cockerell	
Mellitidae	*Hesperapis*	*Hesperapis*	*trochanterata*	Snelling	

Supplementary Table S2. Abundance and diversity measures for bees in three ecosystem types of the Sevilleta National Wildlife Refuge, collected in funnel traps, 2002–2014. "Overall" measures were calculated from data pooled across years, months, and transects within an ecosystem.

	Plains grassland	Chihuahuan Desert grassland	Chihuahuan Desert shrubland
Overall abundance	26,462	26,872	17,617
Mean monthly per-transect abundance	50.89	51.68	33.88
Overall richness	217	207	216
Mean monthly per-transect richness	32.90	31.13	35.50
Overall Shannon H'	2.58	2.11	2.69
Mean monthly per-transect Shannon H'	1.99	1.71	2.08
Overall Pielou's J	0.479	0.395	0.500
Mean monthly per-transect Pielou's J	0.578	0.516	0.592

Supplementary Table S3. Indicator species of each month (March–October) within each of three ecosystem types (Plains grassland, Chihuahuan Desert grassland, and Chihuahuan Desert shrubland), according to Dufrene-Legendre indicator species value. Grey highlights indicate cases in which two or more ecosystem types shared an indicator species in a given month. Asterisks (*) signify cases in which bee species were indicators of different months in different ecosystem types.

	Plains grassland	Chihuahuan Desert grassland	Chihuahuan Desert shrubland
March	*Andrena prima*	*Andrena prima*	*Andrena prima*
	Anthophora porterae	*Anthophora porterae*	*Anthophora porterae*
	Dioxys pacificus	*Dioxys pacificus*	*Dioxys pacificus*
	Eucera lycii	*Eucera lycii*	*Eucera lycii*
	Eucera aff. *albescens*	*Eucera* aff. *albescens*	*Eucera* aff. *albescens*
	Halictus tripartitus	*Halictus tripartitus*	*Halictus tripartitus*
	Melecta pacifica	*Melecta pacifica*	*Melecta pacifica*
	Osmia prunorum	*Osmia prunorum*	*Osmia prunorum*
	Osmia watsoni	*Osmia watsoni*	*Osmia watsoni*
		*Anthidium emarginatum**	*Anthidium emarginatum**
		*Anthophora lesquerellae**	*Anthophora lesquerellae**
		Ashmeadiella erema	*Ashmeadiella erema*
		Osmia phenax	*Osmia phenax*
	Eucera territella	*Eucera territella*	
	Habropoda morrisoni	*Habropoda morrisoni*	
	Osmia aff. *enixa*	*Osmia* aff. *enixa*	
	*Osmia cerasi**	*Osmia cerasi**	
	Osmia liogastra	*Osmia liogastra*	
	Dioxys aff. *pomonae*		*Dioxys* aff. *pomonae*
	*Lasioglossum sisymbrii**	*Andrena mesillae*	*Andrena illinoiensis*
	Osmia (*Melanosmia*) sp. 2	*Atoposmia* aff. *daleae* 2	*Anthidium cockerelli**
	Osmia cordata		*Ashmeadiella rubrella*
	Osmia latisulcata		*Lasioglossum semicaeruleum**
			*Melecta alexanderi**
			*Melecta bohartorum**
April	*Anthophora affabilis*	*Anthophora affabilis*	*Anthophora affabilis*
	Anthophora californica	*Anthophora californica*	*Anthophora californica*
	Anthophora n. sp.	*Anthophora* n. sp.	*Anthophora* n. sp.
	Lasioglossum (*Dialictus*) sp. 2	*Lasioglossum* (*Dialictus*) sp. 2	*Lasioglossum* (*Dialictus*) sp. 2
		Andrena (*Micrandrena*) sp. 2	*Andrena* (*Micrandrena*) sp. 2
		*Lasioglossum sisymbrii**	*Lasioglossum sisymbrii**
		Megachile sublaurita	*Megachile sublaurita*
	Anthidium schwarzi	*Anthidium schwarzi*	
	Dufourea pulchricornis	*Dufourea pulchricornis*	
	*Melecta alexanderi**	*Melecta alexanderi**	

	Plains grassland	Chihuahuan Desert grassland	Chihuahuan Desert shrubland
	Melecta bohartorum*	Melecta bohartorum*	
	Agapostemon angelicus*	Anthidium cockerelli*	Ashmeadiella bucconis
	Anthidium emarginatum*	Atoposmia aff. daleae	Ashmeadiella cactorum
	Anthophora lesquerellae*	Hylaeus episcopelis coquilletti	Dufourea vernalis
	Coelioxys hirsutissima	Lasioglossum semicaeruleum*	Osmia cerasi*
	Dioxys productus	Megachile fucata	
	Lasioglossum hudsoniellum*		
May	Perdita (Perdita) sp. 36	Perdita (Perdita) sp. 36	Perdita (Perdita)sp. 36
		Hoplitis biscutellae	Hoplitis biscutellae
		Perdita coreopsidis kansensis	Perdita coreopsidis kansensis
	Lasioglossum morrilli		Lasioglossum morrilli
	Ceratina nanula	Anthophora montana*	
	Megachile policaris	Colletes clypeonitens	
		Lithurgus apicalis*	
June	Diadasia australis	Diadasia australis	Diadasia australis
	Diadasia rinconis	Diadasia rinconis	Diadasia rinconis
	Anthophora urbana	Agapostemon angelicus*	Lasioglossum (Dialictus) sp. 5
	Colletes scopiventer	Lasioglossum (Dialictus) sp. 7*	Lasioglossum (Dialictus) sp. 8
	Lithurgus apicalis*		Lasioglossum (Dialictus) sp. 13
	Melissodes paroselae		Lasioglossum hudsoniellum*
			Lasioglossum microlepoides*
			Perdita fallax*
July	Halictus ligatus	Halictus ligatus	Halictus ligatus
	Martinapis luteicornis	Martinapis luteicornis	Martinapis luteicornis
	Melissodes tristis	Melissodes tristis	Melissodes tristis
		Ashmeadiella meliloti	Ashmeadiella meliloti
		Diadasia ochracea*	Diadasia ochracea*
		Lasioglossum comulum	Lasioglossum comulum
		Perdita callicerata*	Perdita callicerata*
		Perdita diversa	Perdita diversa
		Perdita ignota ignota*	Perdita ignota ignota*
	Agapostemon melliventris*	Agapostemon melliventris*	
	Lasioglossum aff. pervarum	Lasioglossum aff. pervarum	
	Anthidium porterae*	Anthophorula compactula*	Perdita albovittata
	Lasioglossum (Dialictus) sp. 7*	Lasioglossum (Dialictus) sp. 11	
	Lasioglossum microlepoides*	Lasioglossum lusoria	
	Lasioglossum semicaeruleum*	Neolarra vigilans*	
	Melissodes thelypodii thelypodii	Perdita fallax*	

	Plains grassland	Chihuahuan Desert grassland	Chihuahuan Desert shrubland
	Sphecodes sp. 5		
August	Perdita larreae	Perdita larreae	Perdita larreae
	Perdita marcialis	Perdita marcialis	Perdita marcialis
	Perdita semicaerulea	Perdita semicaerulea	Perdita semicaerulea
		Hesperapis larreae	Hesperapis larreae
		Perdita drymariae	Perdita drymariae
	Anthophora montana*	Anthidium porterae*	Agapostemon melliventris*
	Diadasia ochracea*	Melissodes communis communis	Anthophorula compactula*
	Melissodes coreopsis	Svastra helianthelli	Macrotera portalis*
	Perdita callicerata*		Melissodes agilis*
	Perdita ignota ignota*		Neolarra vigilans*
	Sphecodes sp. 1		Perdita aperta
September		Diadasia diminuta	Diadasia diminuta
		Macrotera latior	Macrotera latior
	Melissodes agilis*	Melissodes agilis*	
	Perdita sp. 13	Anthophorula pygmaea	Anthophora montana*
	Protandrena sp. 2	Macrotera portalis*	Anthophorula completa
			Diadasia megamorpha*
			Macrotera magniceps
October	None	Diadasia megamorpha*	Melissodes snowii
			Perdita austini

Supplementary Table S4. Indicator species for each month (March-October) within the Plains grassland ecosystem, according to Dufrene-Legendre indicator species value. Species are listed from highest to lowest indicator value within each month.

Plains grassland

Species	Family	Indicator value	*P*-value
March			
Osmia watsoni	Megachilidae	1.00	0.001
Eucera lycii	Apidae	0.99	0.001
Anthophora porterae	Apidae	0.99	0.001
Melecta pacifica	Apidae	0.98	0.001
Osmia aff. *enixa*	Megachilidae	0.95	0.001
Eucera aff. *albescens*	Apidae	0.83	0.001
Osmia cerasi	Megachilidae	0.82	0.001
Osmia cordata	Megachilidae	0.80	0.003
Osmia latisulcata	Megachilidae	0.80	0.001
Osmia liogastra	Megachilidae	0.80	0.001
Dioxys pacificus	Megachilidae	0.74	0.001
Andrena prima	Andrenidae	0.60	0.011
Eucera territella	Apidae	0.58	0.002
Osmia (*Melanosmia*) sp. 2	Megachilidae	0.54	0.009
Osmia prunorum	Megachilidae	0.50	0.004
Dioxys aff. *pomonae*	Megachilidae	0.49	0.010
Habropoda morrisoni	Apidae	0.45	0.016
Halictus tripartitus	Halictidae	0.40	0.008
Lasioglossum sisymbrii	Halictidae	0.34	0.002
April			
Dufourea pulchricornis	Halictidae	1.00	0.001
Coelioxys hirsutissima	Megachilidae	1.00	0.001
Anthophora affabilis	Apidae	0.84	0.001
Melecta alexanderi	Apidae	0.78	0.001
Lasioglossum (*Dialictus*) sp. 2	Halictidae	0.77	0.001
Anthidium emarginatum	Megachilidae	0.65	0.001
Melecta bohartorum	Apidae	0.63	0.002
Anthophora n. sp.	Apidae	0.60	0.013
Dioxys productus	Megachilidae	0.60	0.011
Anthophora lesquerellae	Apidae	0.53	0.001
Anthidium schwarzi	Megachilidae	0.52	0.002
Lasioglossum hudsoniellum	Halictidae	0.42	0.001
Anthophora californica	Apidae	0.41	0.014
Agapostemon angelicus	Halictidae	0.22	0.001
May			
Lasioglossum morrilli	Halictidae	0.62	0.001
Perdita (*Perdita*) sp. 36	Andrenidae	0.60	0.006
Ceratina nanula	Apidae	0.60	0.009
Megachile policaris	Megachilidae	0.36	0.050
June			
Diadasia australis	Apidae	0.65	0.001
Lithurgus apicalis	Megachilidae	0.57	0.003
Colletes scopiventer	Colletidae	0.46	0.018
Diadasia rinconis	Apidae	0.42	0.001
Melissodes paroselae	Apidae	0.36	0.038
Anthophora urbana	Apidae	0.34	0.033
July			
Melissodes thelypodii thelypodii	Apidae	0.80	0.001

Martinapis luteicornis	Apidae	0.71	0.002
Anthidium porterae	Megachilidae	0.61	0.001
Agapostemon melliventris	Halictidae	0.56	0.004
Lasioglossum microlepoides	Halictidae	0.47	0.005
Lasioglossum (*Dialictus*) sp. 7	Halictidae	0.37	0.040
Sphecodes sp. 5	Halictidae	0.36	0.046
Halictus ligatus	Halictidae	0.33	0.002
Lasioglossum aff. *pervarum*	Halictidae	0.32	0.002
Melissodes tristis	Apidae	0.32	0.001
Lasioglossum semicaeruleum	Halictidae	0.20	0.023
August			
Perdita semicaerulea	Andrenidae	0.98	0.001
Perdita marcialis	Andrenidae	0.60	0.013
Melissodes coreopsis	Apidae	0.60	0.012
Perdita ignota ignota	Andrenidae	0.60	0.001
Perdita larreae	Andrenidae	0.51	0.012
Sphecodes sp. 1	Halictidae	0.45	0.006
Anthophora montana	Apidae	0.40	0.001
Diadasia ochracea	Apidae	0.38	0.018
Perdita callicerata	Andrenidae	0.36	0.004
September			
Perdita sp. 13	Andrenidae	0.60	0.013
Melissodes agilis	Apidae	0.51	0.003
Protandrena sp. 2	Andrenidae	0.45	0.030
October			
None			

Supplementary Table S5. Indicator species for each month (March-October) within the Chihuahuan Desert grassland ecosystem, according to Dufrene-Legendre indicator species value. Species are listed from highest to lowest indicator value within each month.

Chihuahuan Desert grassland

Species	Family	Indicator value	*P*-value
March			
Eucera lycii	Apidae	0.98	0.001
Osmia watsoni	Megachilidae	0.98	0.001
Anthophora porterae	Apidae	0.96	0.001
Halictus tripartitus	Halictidae	0.96	0.001
Melecta pacifica	Apidae	0.95	0.001
Andrena prima	Andrenidae	0.88	0.001
Osmia phenax	Megachilidae	0.84	0.002
Eucera aff. *albescens*	Apidae	0.80	0.002
Anthidium emarginatum	Megachilidae	0.75	0.001
Osmia aff. *enixa*	Megachilidae	0.73	0.001
Osmia liogastra	Megachilidae	0.70	0.001
Andrena mesillae	Andrenidae	0.60	0.009
Dioxys pacificus	Megachilidae	0.60	0.004
Osmia prunorum	Megachilidae	0.59	0.003
Anthophora lesquerellae	Apidae	0.58	0.001
Osmia cerasi	Megachilidae	0.57	0.004
Ashmeadiella erema	Megachilidae	0.48	0.036
Habropoda morrisoni	Apidae	0.47	0.007
Atoposmia aff. *daleae* 2	Megachilidae	0.45	0.031
Eucera territella	Apidae	0.42	0.015
April			
Anthophora n. sp.	Apidae	0.90	0.001
Anthophora californica	Apidae	0.80	0.001
Melecta alexanderi	Apidae	0.76	0.001
Anthophora affabilis	Apidae	0.76	0.001
Andrena (*Micrandrena*) sp. 2	Andrenidae	0.74	0.001
Megachile sublaurita	Megachilidae	0.66	0.001
Melecta bohartorum	Apidae	0.63	0.001
Hylaeus episcopelis coquilletti	Colletidae	0.60	0.012
Anthidium cockerelli	Megachilidae	0.60	0.013
Megachile fucata	Megachilidae	0.60	0.007
Lasioglossum sisymbrii	Halictidae	0.58	0.001
Dufourea pulchricornis	Halictidae	0.56	0.009
Lasioglossum (*Dialictus*) sp. 2	Halictidae	0.55	0.001
Anthidium schwarzi	Megachilidae	0.53	0.013
Atoposmia aff. *daleae*	Megachilidae	0.45	0.029
Lasioglossum semicaeruleum	Halictidae	0.24	0.003
May			
Hoplitis biscutellae	Megachilidae	0.80	0.001
Perdita (*Perdita*) sp. 36	Andrenidae	0.73	0.003
Colletes clypeonitens	Colletidae	0.60	0.011
Lithurgus apicalis	Megachilidae	0.51	0.015
Perdita coreopsidis kansensis	Andrenidae	0.45	0.001
Anthophora montana	Apidae	0.41	0.013
June			
Diadasia australis	Apidae	0.84	0.001
Diadasia rinconis	Apidae	0.46	0.002
Lasioglossum (*Dialictus*) sp. 7	Halictidae	0.44	0.007
Agapostemon angelicus	Halictidae	0.23	0.009

July			
Lasioglossum (*Dialictus*) sp. 11	Halictidae	0.89	0.001
Lasioglossum comulum	Halictidae	0.74	0.001
Diadasia ochracea	Apidae	0.70	0.002
Perdita ignota ignota	Andrenidae	0.69	0.001
Martinapis luteicornis	Apidae	0.58	0.005
Agapostemon melliventris	Halictidae	0.58	0.001
Halictus ligatus	Halictidae	0.57	0.001
Perdita callicerata	Andrenidae	0.55	0.001
Neolarra vigilans	Apidae	0.48	0.020
Lasioglossum aff. *pervarum*	Halictidae	0.45	0.001
Lasioglossum lusoria	Halictidae	0.42	0.011
Ashmeadiella meliloti	Megachilidae	0.37	0.001
Anthophorula compactula	Apidae	0.37	0.027
Perdita diversa	Andrenidae	0.37	0.029
Melissodes tristis	Apidae	0.34	0.001
Perdita fallax	Andrenidae	0.30	0.024
August			
Hesperapis larreae	Mellitidae	1.00	0.001
Perdita semicaerulea	Andrenidae	0.93	0.001
Perdita drymariae	Andrenidae	0.89	0.001
Perdita marcialis	Andrenidae	0.85	0.001
Perdita larreae	Andrenidae	0.85	0.001
Melissodes communis communis	Apidae	0.71	0.001
Svastra helianthelli	Apidae	0.60	0.011
Anthidium porterae	Megachilidae	0.58	0.001
September			
Macrotera portalis	Andrenidae	0.87	0.001
Anthophorula pygmaea	Apidae	0.80	0.002
Melissodes agilis	Apidae	0.48	0.007
Diadasia diminuta	Apidae	0.40	0.003
Macrotera latior	Andrenidae	0.38	0.034
October			
Diadasia megamorpha	Apidae	0.53	0.003

Supplementary Table S6. Indicator species for each month (March-October) within the Chihuahuan Desert shrubland ecosystem, according to Dufrene-Legendre indicator species value. Species are listed from highest to lowest indicator value within each month.

Chihuahuan Desert shrubland

Species	Family	Indicator value	*P*-value
March			
Osmia phenax	Megachilidae	1.00	0.001
Melecta pacifica	Apidae	1.00	0.001
Eucera lycii	Apidae	0.99	0.001
Anthophora porterae	Apidae	0.98	0.001
Osmia watsoni	Megachilidae	0.96	0.001
Andrena illinoiensis	Andrenidae	0.80	0.002
Melecta alexanderi	Apidae	0.80	0.002
Dioxys aff. *pomonae*	Megachilidae	0.78	0.002
Ashmeadiella erema	Megachilidae	0.74	0.002
Eucera aff. *albescens*	Apidae	0.72	0.003
Anthidium emarginatum	Megachilidae	0.70	0.001
Halictus tripartitus	Halictidae	0.69	0.001
Andrena prima	Andrenidae	0.60	0.006
Ashmeadiella rubrella	Megachilidae	0.60	0.006
Dioxys pacificus	Megachilidae	0.60	0.006
Anthophora lesquerellae	Apidae	0.57	0.001
Melecta bohartorum	Apidae	0.53	0.003
Osmia prunorum	Megachilidae	0.51	0.010
Anthidium cockerelli	Megachilidae	0.50	0.004
Lasioglossum semicaeruleum	Halictidae	0.29	0.001
April			
Anthophora affabilis	Apidae	0.84	0.001
Megachile sublaurita	Megachilidae	0.67	0.001
Dufourea vernalis	Halictidae	0.62	0.003
Ashmeadiella bucconis	Megachilidae	0.60	0.013
Ashmeadiella cactorum	Megachilidae	0.55	0.002
Andrena (*Micrandrena*) sp. 2	Andrenidae	0.55	0.004
Anthophora n. sp.	Apidae	0.54	0.007
Osmia cerasi	Megachilidae	0.48	0.010
Anthophora californica	Apidae	0.42	0.009
Lasioglossum sisymbrii	Halictidae	0.39	0.004
Lasioglossum (*Dialictus*) sp. 2	Halictidae	0.36	0.031
May			
Hoplitis biscutellae	Megachilidae	0.58	0.005
Lasioglossum morrilli	Halictidae	0.48	0.001
Perdita coreopsidis kansensis	Andrenidae	0.41	0.006
Perdita (*Perdita*) sp. 36	Andrenidae	0.36	0.030
June			
Diadasia australis	Apidae	0.83	0.001
Lasioglossum (*Dialictus*) sp. 5	Halictidae	0.60	0.008
Lasioglossum (*Dialictus*) sp. 13	Halictidae	0.60	0.005
Lasioglossum microlepoides	Halictidae	0.47	0.001
Diadasia rinconis	Apidae	0.44	0.001
Lasioglossum (*Dialictus*) sp. 8	Halictidae	0.38	0.033
Perdita fallax	Andrenidae	0.31	0.027
Lasioglossum hudsoniellum	Halictidae	0.31	0.002
July			
Martinapis luteicornis	Apidae	0.77	0.001

Diadasia ochracea	Apidae	0.73	0.001
Lasioglossum comulum	Halictidae	0.63	0.004
Perdita ignota ignota	Andrenidae	0.44	0.006
Perdita callicerata	Andrenidae	0.43	0.001
Perdita diversa	Andrenidae	0.42	0.011
Halictus ligatus	Halictidae	0.41	0.004
Perdita albovittata	Andrenidae	0.40	0.036
Ashmeadiella meliloti	Megachilidae	0.30	0.009
Melissodes tristis	Apidae	0.25	0.001
August			
Hesperapis larreae	Mellitidae	0.99	0.001
Perdita larreae	Andrenidae	0.94	0.001
Perdita semicaerulea	Andrenidae	0.88	0.001
Perdita marcialis	Andrenidae	0.81	0.001
Perdita drymariae	Andrenidae	0.65	0.003
Macrotera portalis	Andrenidae	0.61	0.002
Perdita aperta	Andrenidae	0.60	0.009
Anthophorula compactula	Apidae	0.57	0.001
Neolarra vigilans	Apidae	0.46	0.009
Melissodes agilis	Apidae	0.38	0.026
Agapostemon melliventris	Halictidae	0.33	0.027
September			
Macrotera latior	Andrenidae	0.45	0.010
Macrotera magniceps	Andrenidae	0.45	0.039
Diadasia diminuta	Apidae	0.41	0.001
Anthophora montana	Apidae	0.39	0.002
Diadasia megamorpha	Apidae	0.36	0.038
Anthophorula completa	Apidae	0.33	0.041
October			
Melissodes snowii	Apidae	0.40	0.023
Perdita austini	Andrenidae	0.34	0.042

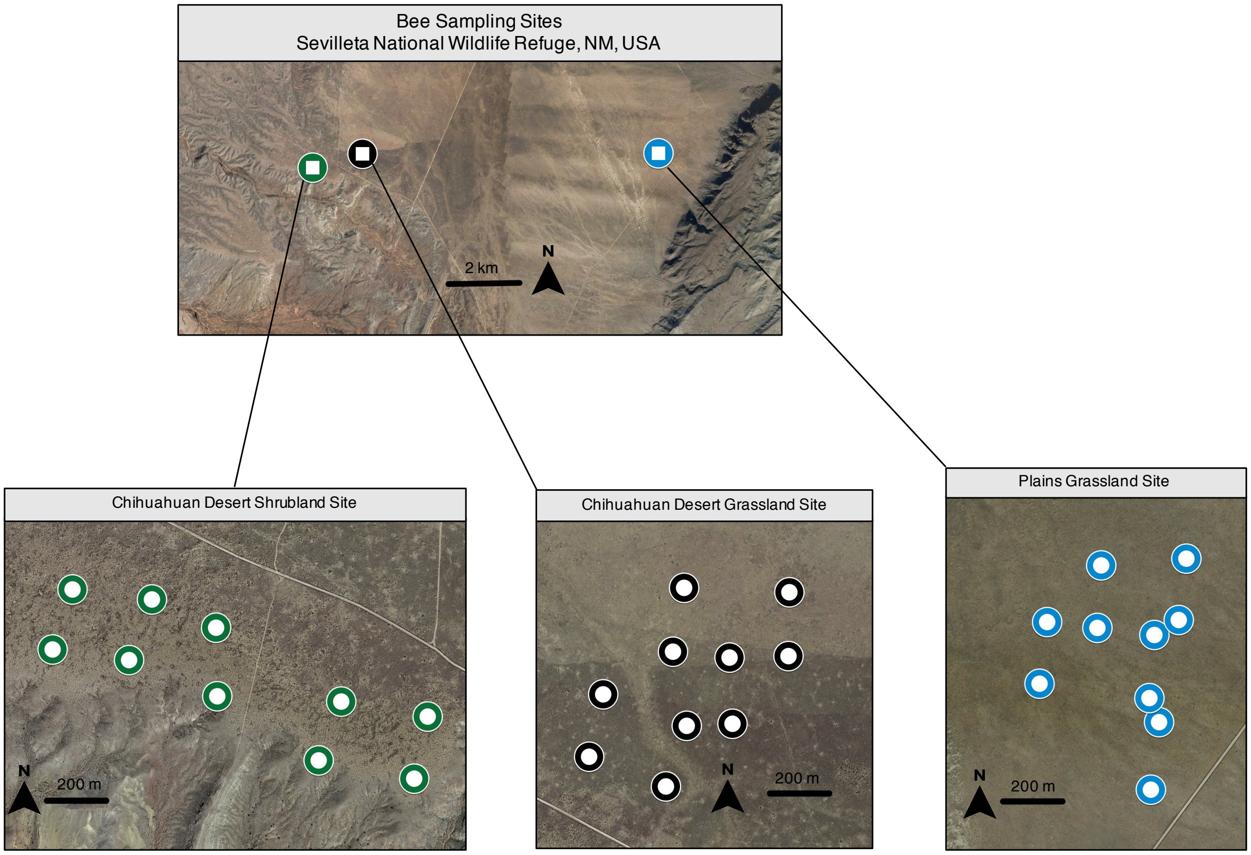

Supplementary Figure S1. Map of sampling sites at the Sevilleta National Wildlife Refuge, NM, USA. Bees were sampled in three focal ecosystem types: Chihuahuan Desert shrubland, Chihuahuan Desert grassland, and Plains grassland. To sample bees, we installed one passive funnel trap at each end of five 200 m transects/site; traps are indicated by colored points on the three lower panels. Maps were generated via Google Maps (https://maps.google.com; map imagery: TerraMetrics, 2019).

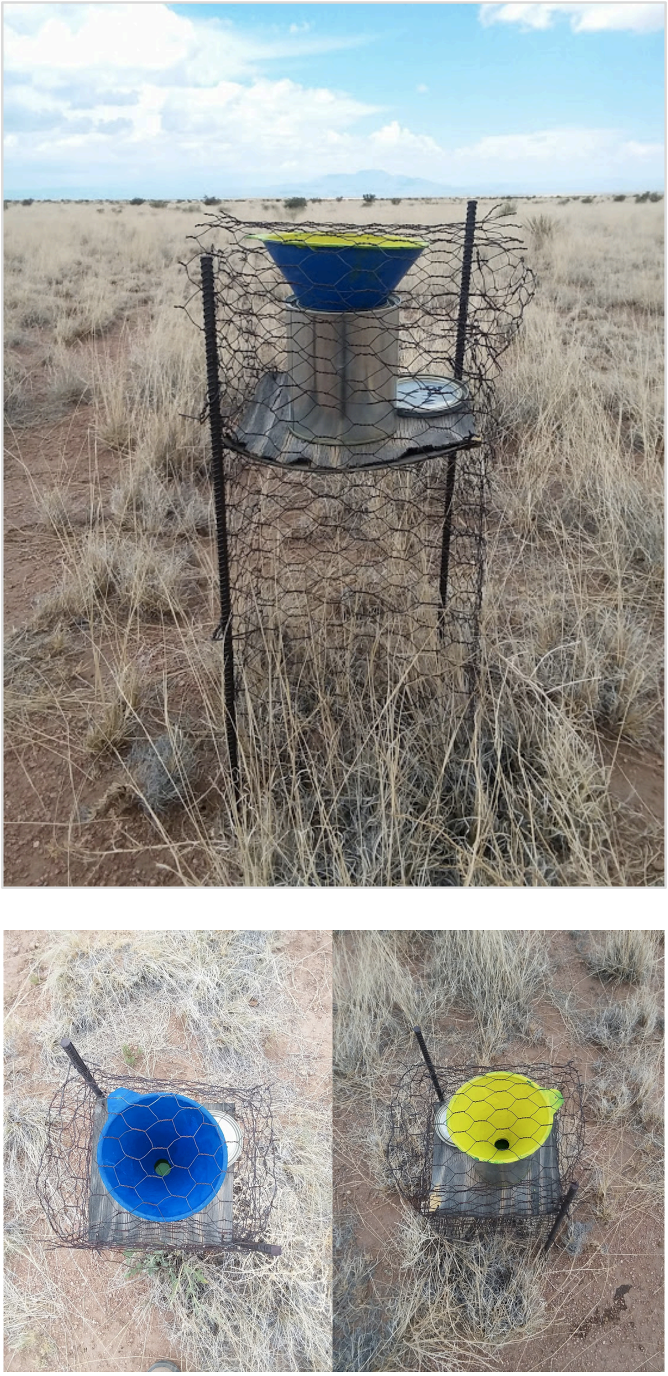

Supplementary Figure S2. Funnel traps used for bee collection. Each trap consisted of a 946 mL paint can filled with ~275 mL of propylene glycol and topped with a plastic automotive funnel (funnel height = 10 cm, top diameter = 14 cm, bottom diameter = 2.5 cm). The funnels' interiors were painted with either blue or yellow fluorescent paint (Krylon, Cleveland, OH or Ace Hardware, Oak Brook, IL). Each trap was placed on a 45 cm high platform that was surrounded by a 60 cm high chicken wire cage to prevent wildlife and wind disturbance.

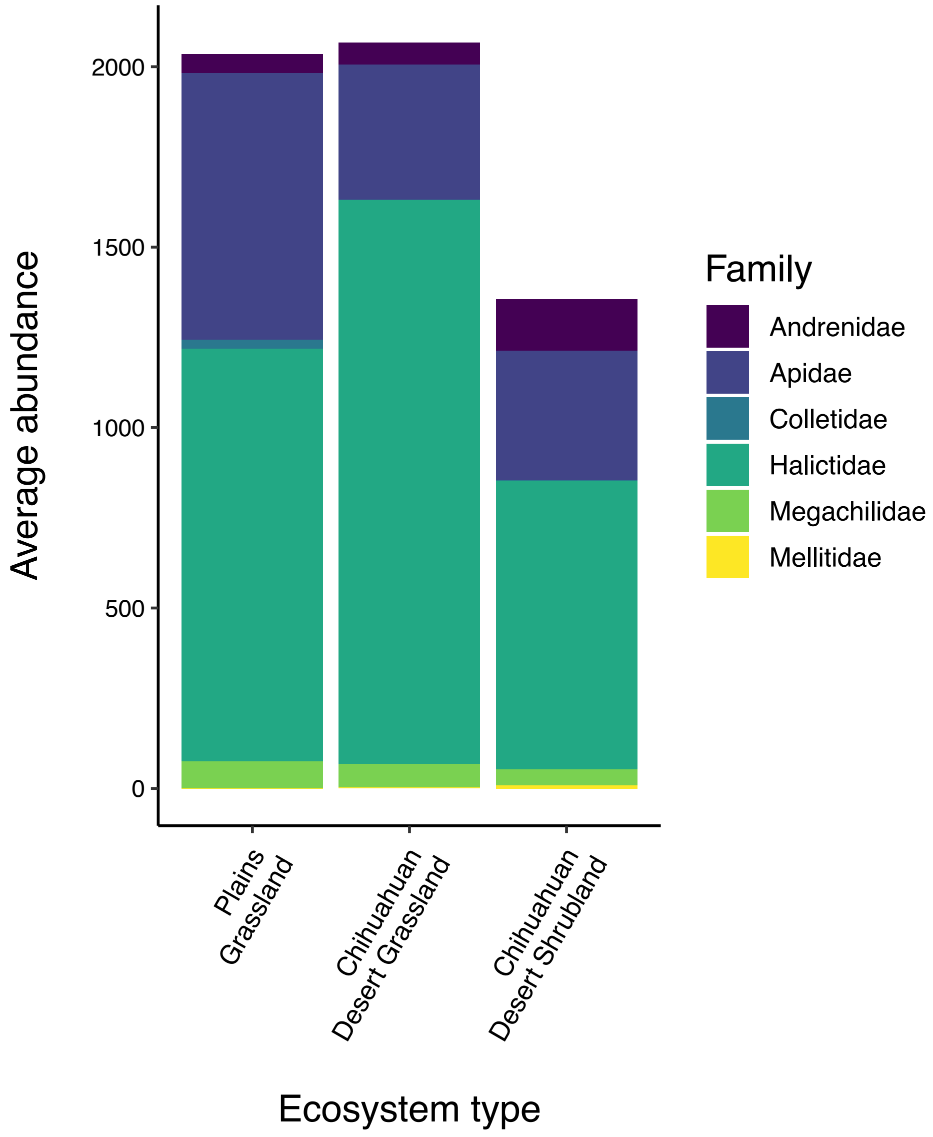

Supplementary Figure S3. Average yearly abundance of bees by family in each of the three studied southwestern U.S. ecosystem types.

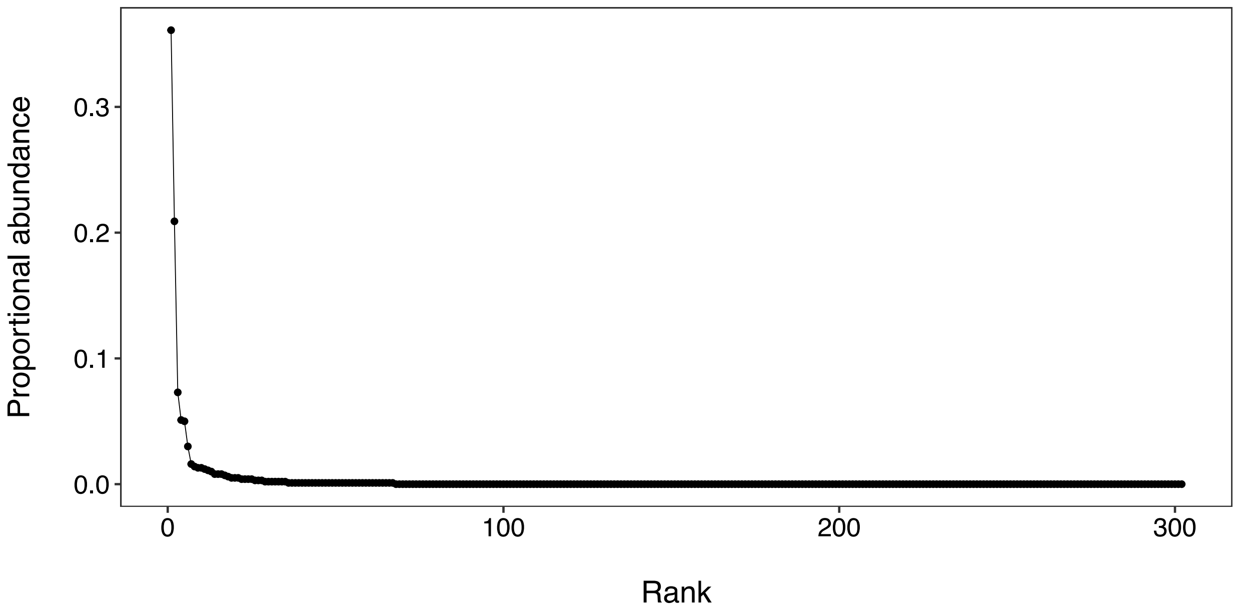

Supplementary Figure S4. Proportional abundance by abundance rank for the 302 bee species collected across three southwestern U.S. ecosystem types during the study.

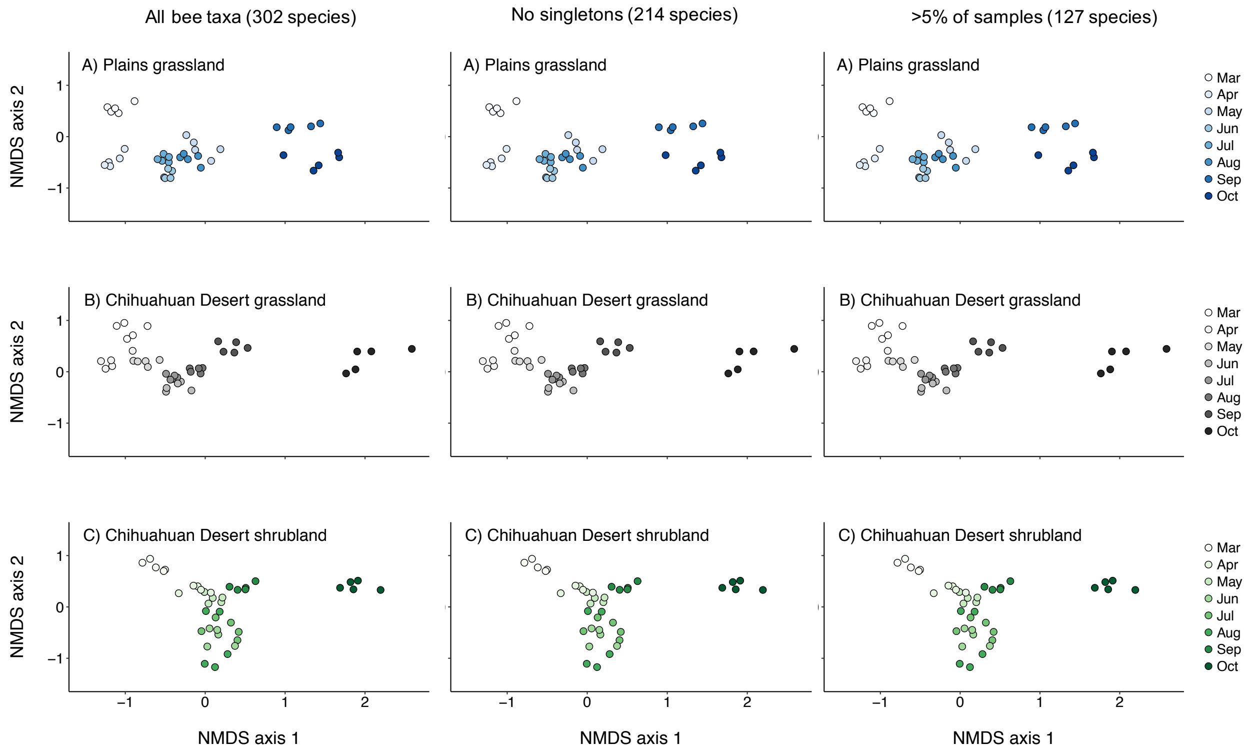

Supplementary Figure S5. Non-metric multidimensional scaling (NMDS) plots depicting variation in bee species composition for three dryland ecosystem types: (A) Plains grassland, (B) Chihuahuan Desert grassland, and (C) Chihuahuan Desert shrubland. NMDS analyses were run separately for the full dataset (left column), the dataset with singleton bee species removed (center column), and the dataset with only bee species present in >5% of samples (right column).

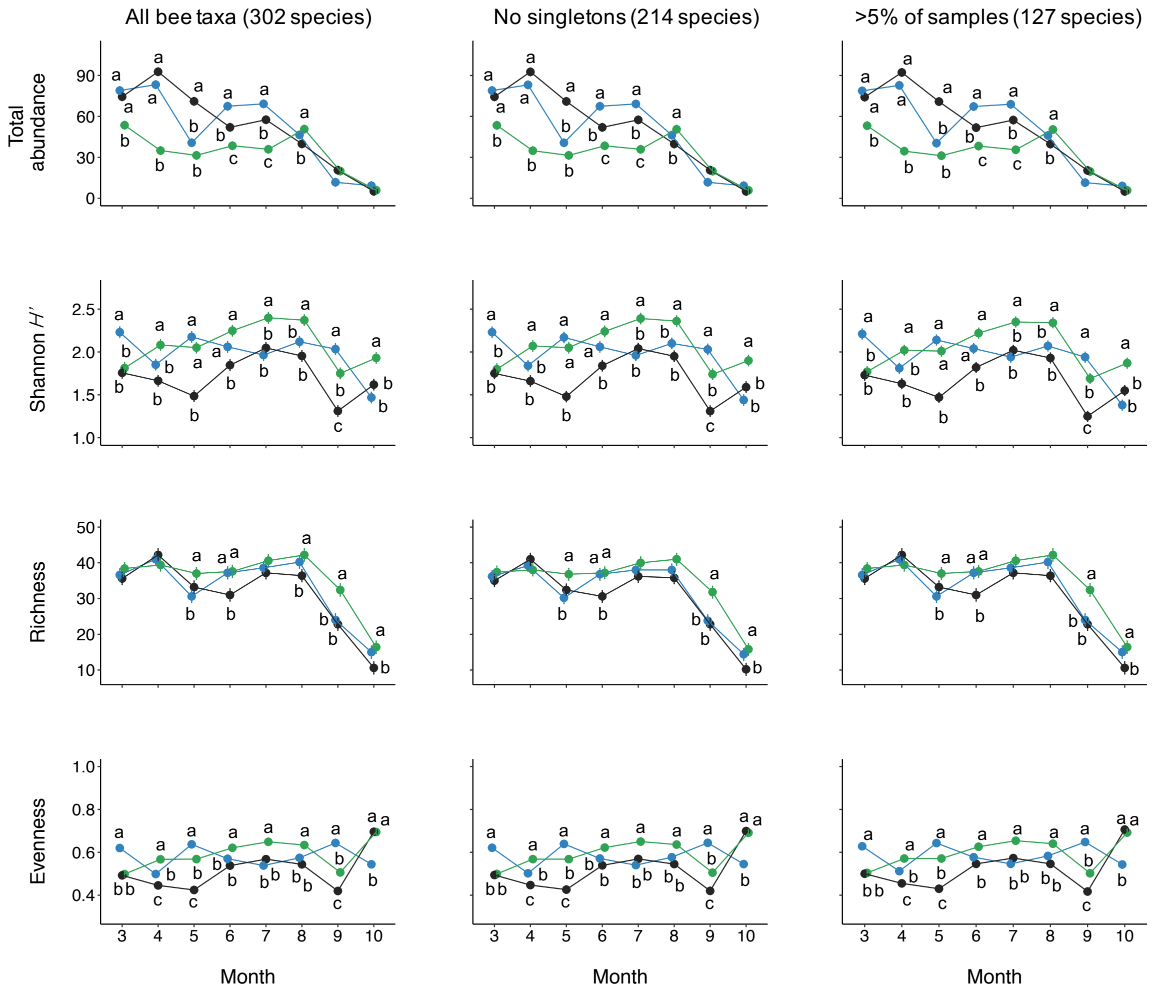

Supplementary Figure S6. Variation across sampling months in per-transect bee abundance, Shannon diversity (*H'*), richness, and evenness (Pielou's *J*), ± s.e., for three dryland ecosystems: Plains grassland (blue points), Chihuahuan Desert grassland (black points), and Chihuahuan Desert shrubland (green points). Analyses were run for the full dataset (left column), the dataset with singleton bee species removed (center column), and the dataset with only bee species present in >5% of samples (right column). Letters denote contrasts between ecosystems within a given month; ecosystems labeled with different letters differed significantly from one another in the relevant abundance/diversity metric. Points lacking letters did not differ significantly from any other ecosystem in the given month.
